# 

**Published:** 1889-03-30

**Authors:** 


### IMPORTANT NOTICE.

The First Chapter of

### A NEW SERIAL STORY

ENTITLED

### Her Heart's Mistake,

By E. D. CUMMING.

*Author of "An Ordeal by Silence," etc.*

Will be commenced in our next issue, being No. 1 of Volume VI.

### A ROYAL NUMBER

Of The Hospital will be published on April 20th, 1889. It will contain an Account of the Public Life and Work of their Royal Highnesses

THE PRINCE AND PRINCESS OF WALES.

⁂ The monthly part for May will contain Special Portraits of their Royal Highnesses. See notice below as to separate issue.

Intending Subscribers should send their names to the publisher, The Hospital, 139, Salisbury Court, London, E.C., without delay.

### OUR NEW OFFICES,

### 139 AND 140, SALISBURY COURT, FLEET STREET, E.C.

Our readers are asked to note that with the new volume (which begins on April 6th) portraits of leaders in the medical, philanthropic and nursing world will be issued with the monthly parts. For the convenience of weekly subscribers, *these portraits will be issued separately, at 3d. each, but must be ordered at an early date* to secure prompt delivery on date of publication. In consequence of the growing requirements of The Hospital, and of the inconvenience to the trade arising from the distance of our present offices from the publishing centre,

### ON AND AFTER APRIL 1st

The Hospital will be issued from, and all advertising and other communications should be sent to Our New Offices, 139 and 140, Salisbury Court, Fleet Street, E.C.

### Scraps and Gleanings.

Lord Leigh presided at the annual meeting of Warneford Hospital.

The extension of Fulham Union has been commenced. The cost will be £17,000.

It is proposed to receive paying patients at the Manchester Eye and Ear hospital.

Mr. Greswolde-Williams has sent a cheque for £500 to the Worcester Infirmary Committee.

Dr. Smith is delivering a series of ambulance lectures to the Midland Railway staff at St. Pancras.

Captain Wrangham has given £400 to the Children's Ward of the Princess Alice Memorial Hospital.

Dr. Kenneth Millican and Dr. Lindsay Johnson have joined the medical staff of the West-end Hospital.

The death is announced of Dr. John Boutflower, for forty years connected with Salford Royal Hospital.

During last year the *Times* recorded the deaths of 193 persons over 90 years of age; 125 of them were women.

Watlington Cottage Hospital annual report is issued and is very satisfactory. There is a balance of £34.

Mr. T. W. Wing, lately deceased, has left a large sum of money to be distributed to blind persons as annuities.

The Metropolitan Asylums Board estimate their expenses for the half-year ending Michaelmas next at £173,698.

Over a hundred thousand penny dinners have been given to poor children in Manchester during the winter.

Dr. J. Poole has been elected physician to the Ulster Hospital for Women and Children, after a close contest.

Dr. Mueller, of Yackandandah, says he has found injections of liq. strychnine a cure for poison by snake-bites.

At the triennial festival of the Royal General Dispensary, last week, contributions to the amount of £400 were announced.

Mr. E. Reynolds, an artist, died last week from the effects of solution of sulphate of mercury, which he drank by mistake.

Last Sunday the Bishop of Leicester preached an excellent sermon at the Chapel Royal, Whitehall, in aid of the Cancer Hospital.

The last Registrar-General's report gives as the six healthiest places Sunderland, Oldham, Leicester, Cardiff, Derby, and Birmingham.

Fairford Cottage Hospital treated fifty-seven patients last year. Total expenditure, £201, of which £4 was received from the patients.

Dr. Wolfe, of Glasgow, has been presented, on the occasion of his marriage, with a piece of silver plate from his professional brethren.

Leeds Infirmary has received £7,000 by the recent death of Mr. Thomas Tennant; the Hospital for Women and Children, Leeds, receives £1,000.

Dr. M'Kellar, for eighteen years medical superintendent of the Metropolitan Board's hospitals, is to be presented with £1,000 on his resignation.

St. Bartholomew's Hospital, Chatham, has greatly increased its efficiency by establishing separate wards for the treatment of medical and surgical cases.

The published reports, it is officially said, greatly exaggerate the number of deaths from yellow fever at Rio de Janeiro. The daily average of deaths is 15.

The late Mr. Robert Paterson, merchant, Glasgow, has bequeathed about £35,000 to the principal charities of that city, including £10,000 to the Western Infirmary.

Birkenhead has a homœopathic mayor, and he waxed eloquent in defence of his theories at the late meeting of the subscribers to the Homœopathic Dispensary.

Mr. J. Crossley, a large employer of workmen in Ripley, has offered to pay £20 per annum for four or five years as rent for a suitable building as a cottage hospital for Ripley.

The Royal Eye Hospital, Manchester, has lost an old friend by the death of Alderman Goldschmidt. The Board has passed a resolution of sympathy with the bereaved family.

A poor woman in the Government of Kazan, named Balbura Mamakoff, is said to have lately been delivered of five children, all boys, at a birth. Only three are alive and well.

Dr. Fox has brought before the Winsford Local Board a case of a man who contracted scarlet fever from a book belonging to the free library, which had been in a house where fever was rife.

Professor Donders, of Flanders, the famous oculist, died lately at the age of seventy-one. He was a Doctor *honoris causa* of Utrecht University. He was also a member of many foreign and English Academies.

The free dinners instituted by Princess Christian for the poor children of Windsor have just ceased. The meals were provided twice a week during the winter season. No fewer than 5,569 dinners were given, the average attendance of children at each meal being 242.

The commission for considering the feasibility of establishing a teaching university in London has, it is believed, agreed upon the basis of its report. No encouragement will be given to the request of the College of Physicians and the College of Surgeons that they should be allowed to grant degrees.

John Kynaston, who was convicted of contravening the Lunacy Laws by receiving into his house, which was not licensed, a female lunatic named Laura Louisa Corlett, was brought up for sentence at the Liverpool Assizes. Mr. Justice Charles said he did not find it his duty to send the defendant to prison, but the offence was a serious one, and he must inflict a penalty of £100.

The enlargement at the Princess Alice Memorial Hospital, Eastbourne, from Mr. Cutler's plans, includes two large wards. Each of the two wards measures about 70 feet by 25 feet, and the cubical contents give 1,300 feet per bed and a floor space of 88 feet. The windows give 22 feet 7 inches of superficial light to each bed, and, when open, will admit air through apertures of 12 feet 9 inches per bed.

### Amusements and Relaxation.

### NEW PUZZLE PRIZE SCHEME.

SECOND QUARTERLY COMPETITION COMMENCED JANUARY 5TH, ENDS MARCH 30TH, 1889.

1. Prizes will be awarded for the best Original Puzzles in Prose or Verse relating to any of the Social, Political, or Philanthropical topics of the day. Competitors are requested to *strictly* observe this rule, or their contributions will be discredited.

2. Puzzles may take any form which the ingenuity of the competitors suggest. Answers must accompany contributions, and the source of obscure or ambiguous terms be given.

3. No competitor will be allowed to take more than one prize during the Quarter.

4. Eight Prizes of Standard Books will be given, the choice of the book to be left to the winner. First, Second, Third, Fourth, and Fifth Prizes for best Original Puzzles, value £1 1s., 15s., 10s. 6d., 7s. 6d., and 5s. each.

5. First, Second, and Third Prize for Solutions of 15s., 10s. 6d., and 5s. each.

N.B.—All contributions must reach the office, 38, Old Jewry, E.C., not later than the *First Post* on *Thursday* in each week.

### ORIGINAL PUZZLES FOR SOLUTION.

**Thirteenth Week.**

No. 26. Double Acrostic.

Students of the art of healing
Thankful be to these great minds!
Whose patient thought with science dealing,
Them ever to our memory binds.

1. What anguish fills his soul as he draws near!
He now must give the thing he holds most dear.
2. Thy pen hath often charmed us with its skill,
Thy clever works our hours with pleasure fill.
3. Look in the "Doctor's Pulpit" and in the Treasurer's plea,
Or search throughout The Hospital. this word will never be.
4. Close to where Eastern waters ebb and rise,
South of an empire vast, this small town lies,
5. The best of much in little this light does here disclose
The value of its use in food our worthy doctor knows.
6. A freebooter of fame on northern ground this man,
Who ever tried through his exploits rebellion's fire to fan.

Cotonopolis.

No. 27. Arithmorem.

| | |
|---|---|
| 552 and a | A Greek epic poem. |
| 1001 and R | A border or margin. |
| 510 and eous | A Book of the Old Testament. |
| 550 and an | Earth. |
| 1050 and sa | Charity to the poor. |
| 152 and not | Relating to the Nile. |
| 551 and nge | A seaport town in Ireland. |

The initials will give a country at present much disturbed. Nipper.

**Marks for Original Puzzles.**

| Names. | No. of Puzzles sent in Mar. 21. | No. of Marks Mar. 21. | Totals. | Names. | No. of Puzzles sent in Mar. 21. | No. of Marks Mar. 21. | Totals. |
|---|---|---|---|---|---|---|---|
| Cotonopolis... | 1 | 2 | 45 | Prior........... | 3 | 4 | 26 |
| Tinie............ | 3 | 4 | 54 | Lightowlers . | 3 | 3 | 30 |
| Jenny Wren . | 3 | 3 | 7 | Twentieth ... | — | — | 6 |
| Patience ...... | 3 | 5 | 63 | Crocodile...... | — | — | 14 |
| Padre ......... | — | — | 3 | Nabob ......... | 1 | 3 | 17 |

**Answers.**

No. 23. Double Acrostic.

1 L aure L

2 E ffend I

3 N umera L

4 T apestr Y

No. 24. Diamond Puzzle.

```
      G
    B E E
  A R R O W
G E R M A N Y
  S P A R K
    A N T
      Y
```

**Marks for Solution of Puzzles.**

Total Marks.—Flora (2), 12; Mater (1), 9; Scrooge (2), 12; Patience, 4; Lady Clara (2), 13; Ruffles (2), 9; Lauriston, (2), 3; Reldas (1), 9; Jenny Wren (1), 5; Condorcet 2; Tinie, 2; Daffodil (I), 1.

[*The Figures in parentheses are Marks for the week ending March 21st.*]

### SPECIAL NOTICE.

A New Orginal Acrostic Competition in verse will be commenced in these columns the first week in April.

Acrostics to be sent in every fortnight, commencing Thursday, April 4th, and ending June 27th.

Two prizes will be given—First Prize of One Guinea, and Second Prize of 10s. 6d.

Acrostics will be credited according to merit, six marks being the highest score given for any one Acrostic.

In addition to the above prizes, one of *Half-a-Guinea* will be given for Solution of Acrostics set.

One mark only will be given for the correct solution of all lights of each Acrostic.

**Word Competition.**

At the request of many of our readers we have decided to start another weekly Word Dissection, commencing April 4th, and ending July 4th.

Three Prizes of 15s., 10s., 5s., will be given for the largest number of words derived from the word set for dissection.

Proper names, abbreviations, foreign words, words of less than four letters, and repetitions are barred; plurals, and past and present participles of verbs, are allowed. Nuttall's *Standard* dictionary only to be used.

N.B.—All words sent in must be arranged *alphabetically*, with correct total affixed.

(For Conditions see The Hospital, March 16th.)

